# Elderly-Onset Brainstem Myelin Oligodendrocyte Glycoprotein Antibody-Associated Disease: A Glioma Mimic

**DOI:** 10.7759/cureus.117898

**Published:** 2026-10-14

**Authors:** Niveditha Apparasu, Anudeep Surendranath, Abhishek Kengen, Anusha Reddy Muddasani, Yashwanth K Pingali

**Affiliations:** 1 Internal Medicine, Kamineni Academy of Medical Sciences and Research Centre, Hyderabad, IND; 2 Neurology, CHI St. Vincent Hot Springs, Hot Springs, USA; 3 Neurology, CHI Memorial Hospital, Chattanooga, USA; 4 Internal Medicine, University of Arkansas, Fayetteville, USA; 5 Radiodiagnosis, Chalmeda Anand Rao Institute of Medical Sciences, Karimnagar, IND

**Keywords:** brainstem lesions, elderly-onset demyelination, glioma mimic, mog-igg titers, myelin oligodendrocyte glycoprotein antibody-associated disease (mogad)

## Abstract

Myelin oligodendrocyte glycoprotein antibody-associated disease (MOGAD) is an autoimmune inflammatory demyelinating disorder of the central nervous system with a broad clinical spectrum, although brainstem-predominant presentations in the elderly are rare and may closely mimic neoplastic lesions. We report a case of an 81-year-old female who presented with progressive gait imbalance, binocular diplopia, right-sided sensory symptoms, and mild headache. Magnetic resonance imaging demonstrated an expansile contrast-enhancing pontine lesion extending to the pontomesencephalic junction, initially raising strong suspicion for glioma. Evaluation for infectious, neoplastic, and autoimmune etiologies was unrevealing except for low-positive (1:32) serum myelin oligodendrocyte glycoprotein immunoglobulin G (MOG-IgG), supporting the diagnosis of MOGAD. The patient was treated with high-dose intravenous corticosteroids, resulting in marked clinical improvement. Two early symptomatic recurrences after corticosteroid treatment led to initiation of mycophenolate mofetil. Follow-up imaging demonstrated near-complete resolution of the lesion on T2-weighted fluid-attenuated inversion recovery imaging, with complete resolution of contrast enhancement. This case emphasizes that MOGAD should be included in the differential diagnosis of atypical enhancing brainstem lesions in older adults, as early antibody testing and prompt immunotherapy may help avoid misdiagnosis and unnecessary neurosurgical or oncological interventions.

## Introduction

Myelin oligodendrocyte glycoprotein (MOG), a member of the immunoglobulin superfamily, is located on the outer surface of myelin sheaths and oligodendrocyte membranes, where it is accessible to circulating antibodies. Cell-based assays using full-length, conformationally intact MOG have detected MOG antibodies in subsets of patients, including children with acute disseminated encephalomyelitis (ADEM), patients with aquaporin-4 (AQP4)-seronegative neuromyelitis optica spectrum disorder (NMOSD), and those with monophasic or recurrent optic neuritis (ON) or myelitis. These antibodies are often transient after a single episode, such as ADEM, but may persist in multiphasic ADEM, NMOSD, and recurrent ON or myelitis. Biopsy studies have described demyelination resembling that seen in multiple sclerosis (MS), with oligodendrocyte pathology and antibody and complement involvement. As of a 2017 review, the direct pathogenic role of these antibodies and the status of antibody-positive patients as a distinct disease entity remained under investigation [1].

Scientific interest in MOG dates back to the 1980s, when the M2 antigen, a glycoprotein component of central nervous system (CNS) myelin expressed on oligodendrocytes, was identified as a target of autoantibodies [2]. M2 was subsequently shown to be identical to MOG [1]. In 2003, serum IgM antibodies against MOG and myelin basic protein (MBP), detected by Western blot, were proposed as biomarkers for predicting conversion from clinically isolated syndrome to MS [3]. However, a larger study of 462 patients with clinically isolated syndrome found that antibodies against MOG and MBP did not predict progression to MS [4], and anti-MOG antibodies measured with an earlier radiobinding assay were found at similarly low frequencies in patients with MS and healthy controls, indicating that they were not disease-specific [5].

In 2007, O’Connor et al. developed an assay that distinguished antibodies against native, correctly folded MOG from those against denatured MOG; antibodies to native MOG were found in a subset of patients with ADEM but only rarely in adult-onset MS [6]. Later, mammalian cell-based assays expressing full-length MOG detected MOG immunoglobulin G (MOG-IgG) in 18% of children with acquired demyelinating syndromes, mostly those with an ADEM-like onset [7], and in 6.3% of adults with inflammatory demyelinating disease, in whom it identified a phenotype distinct from NMOSD and MS [8]. A systematic review and meta-analysis of 14 studies found MOG-IgG positivity in 32.5% of AQP4-IgG-seronegative neuromyelitis optica (NMO)/NMOSD cases, rising to 41.6% when only seronegative cases diagnosed by the 2015 International Panel for NMO Diagnosis criteria were included [9].

MOG antibody-associated disease (MOGAD) has a broad spectrum of clinical and radiological presentations, extending beyond the optic nerve and spinal cord [10]. Its manifestations may overlap with those of ADEM and NMOSD [11,12]. MOGAD can follow either a monophasic or relapsing course [12]. Compared with AQP4-IgG-positive NMOSD, it is generally associated with fewer attacks and better functional recovery [13], although relapses and persistent disability can occur [12,14]. MOGAD is considered immunopathogenetically distinct from both MS and AQP4-IgG-positive NMOSD, and some therapies used for MS may be ineffective or even harmful in MOGAD [14,15]. Acute MOGAD attacks are generally treated with corticosteroids, with plasma exchange or intravenous immunoglobulin (IVIG) as additional or alternative options [14]. For patients with relapsing disease, maintenance options include oral corticosteroids, IVIG, rituximab, and mycophenolate mofetil, although relapses can occur despite treatment [14,16].

Age at onset may influence the clinical presentation and outcomes of inflammatory demyelinating disorders. In MS, late-onset disease has been associated with a less favorable long-term prognosis [17,18]. In late-onset NMOSD (onset at 50 years or older), outcomes have been characterized mainly by motor disability with relatively preserved visual function, and older age at onset has been associated with mortality [19]. In children with acquired demyelinating syndromes, MOG-IgG is frequently detected, particularly in those with ADEM [7,20,21]. Isolated brainstem or encephalopathic presentations are uncommon in adults with MOGAD, accounting for 6.6% of onset presentations in a large French cohort [10]. Recognition of such presentations in older adults is particularly important because brainstem lesions may raise concern for neoplastic disease.

## Case presentation

An 81-year-old female with a medical history of hyperlipidemia, paroxysmal atrial fibrillation, mild diastolic dysfunction, hypothyroidism, and peptic ulcer disease presented with a three-day history of progressive gait imbalance, binocular diplopia, and right-sided sensory disturbances. She had been living independently and had no relevant family history of neurological disease, tobacco or alcohol use, or recent infectious illness.

Her symptoms began with unsteadiness while walking, which she described as “walking like a drunk,” followed by transient numbness of the right calf and leg. Over the next 48 hours, she developed binocular horizontal diplopia that was more pronounced with distance vision and resolved when either eye was covered, consistent with a binocular ocular misalignment. One day before admission, she noted numbness involving the right forearm, hand, and right side of the tongue, accompanied by a mild headache and difficulty performing fine motor tasks such as buttoning clothing.

On presentation, vital signs were stable. Despite her subjective complaints, the neurological examination was largely nonfocal. Cranial nerve function was intact, extraocular movements were full without obvious ophthalmoplegia, motor strength was 5/5 throughout, deep tendon reflexes were symmetric, and coordination and sensory testing were normal. No cerebellar signs were elicited during bedside examination.

Brain magnetic resonance imaging (MRI) with gadolinium demonstrated an expansile T2-weighted fluid-attenuated inversion recovery (T2-FLAIR) hyperintense lesion involving the upper pons with extension to the pontomesencephalic junction, associated with mild mass effect (Figures 1, 2). Post-contrast T1-weighted axial and coronal images demonstrated ill-defined patchy foci of enhancement within the left hemipons (Figures 3, 4). These findings initially raised suspicion for a brainstem glioma.

**Figure 1:**
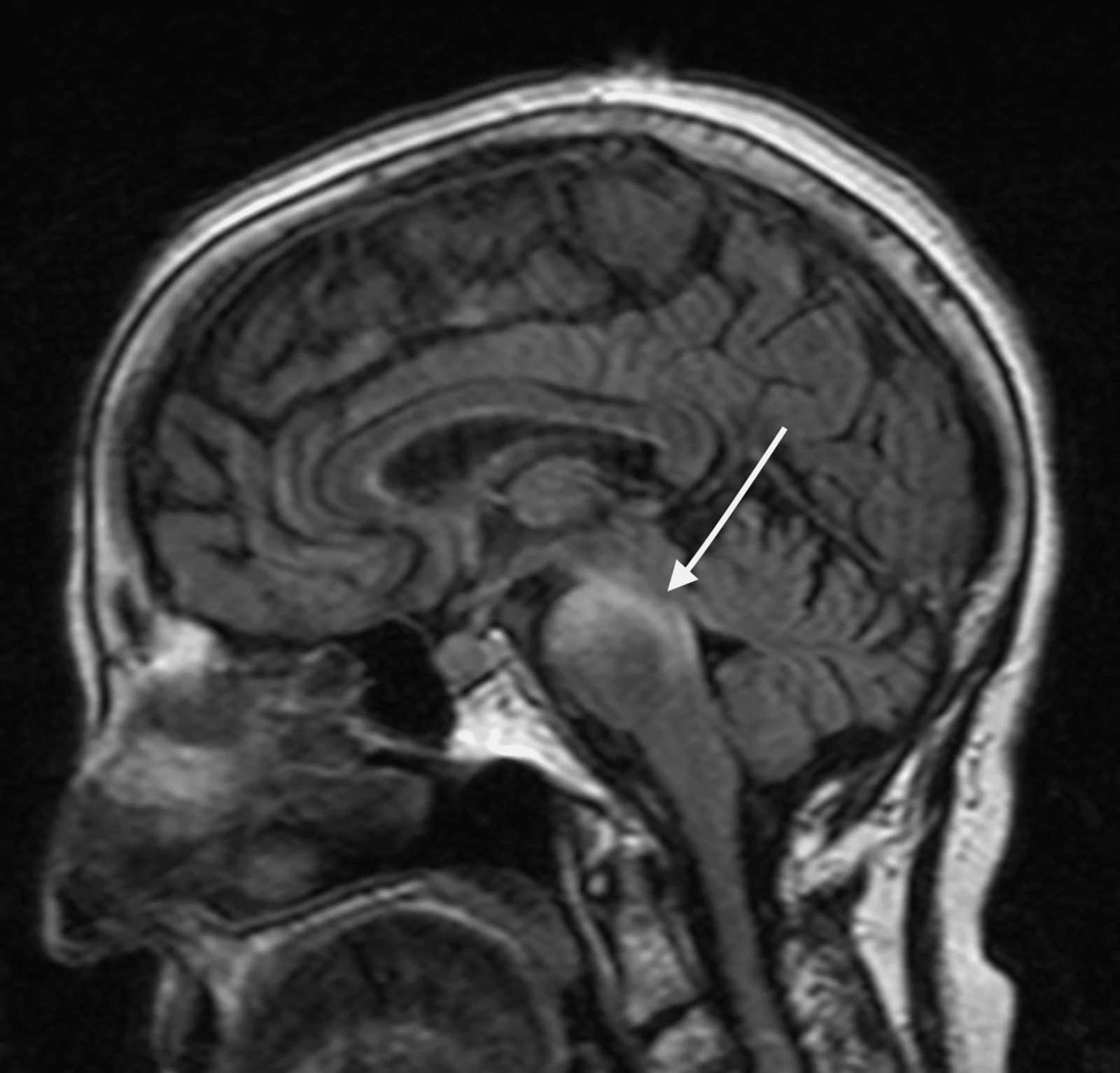
Sagittal T2-weighted fluid-attenuated inversion recovery (T2-FLAIR) MRI demonstrating an ill-defined hyperintense lesion within the upper pons, extending superiorly to the pontomesencephalic junction. The arrow indicates the upper pontine hyperintense lesion.

**Figure 2:**
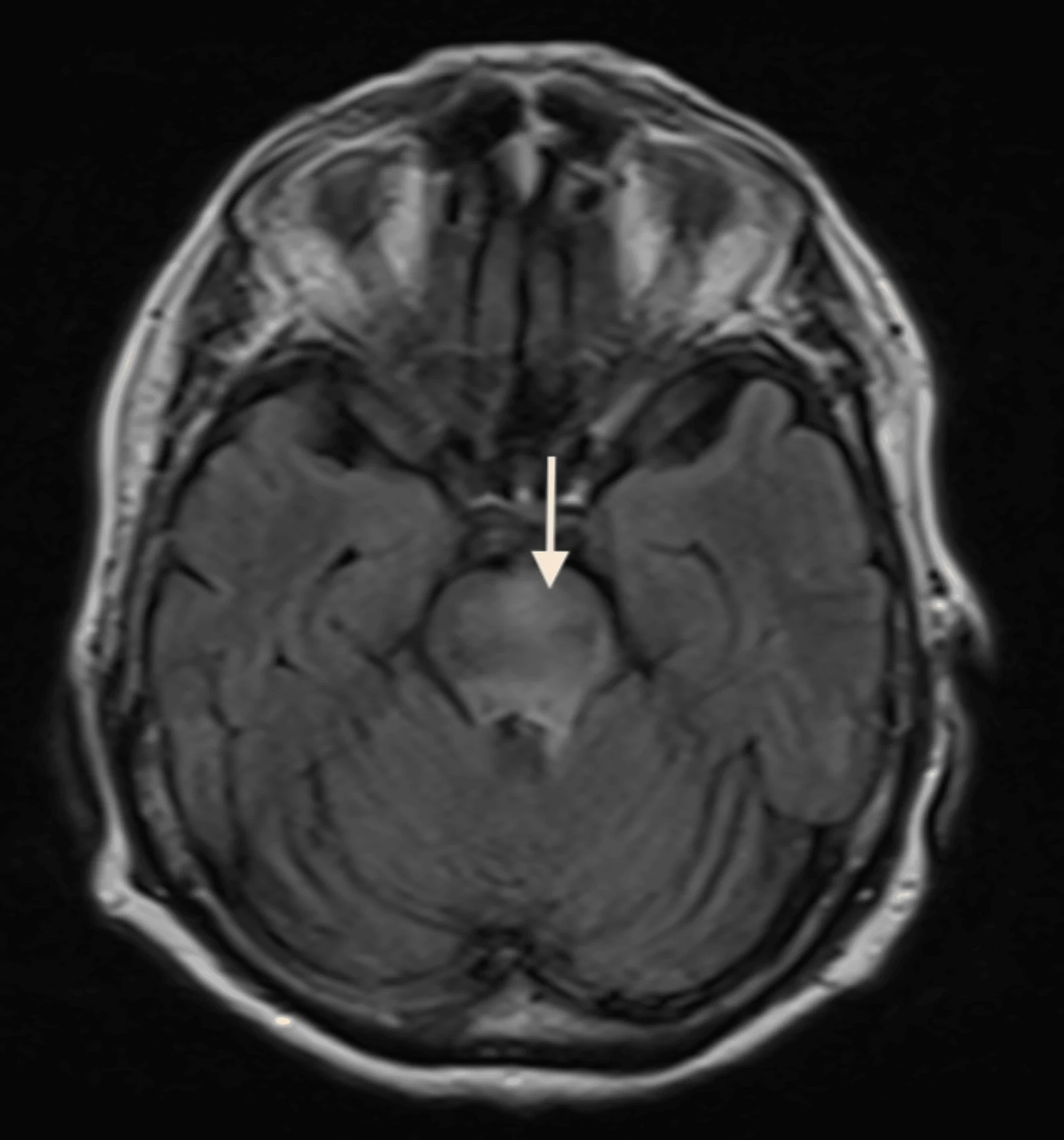
Axial T2-weighted fluid-attenuated inversion recovery (T2-FLAIR) MRI demonstrating an ill-defined, patchy hyperintense lesion involving the pons, predominantly affecting the left hemipons and bilateral superior cerebellar peduncles. The arrow indicates the left pontine hyperintense lesion.

**Figure 3:**
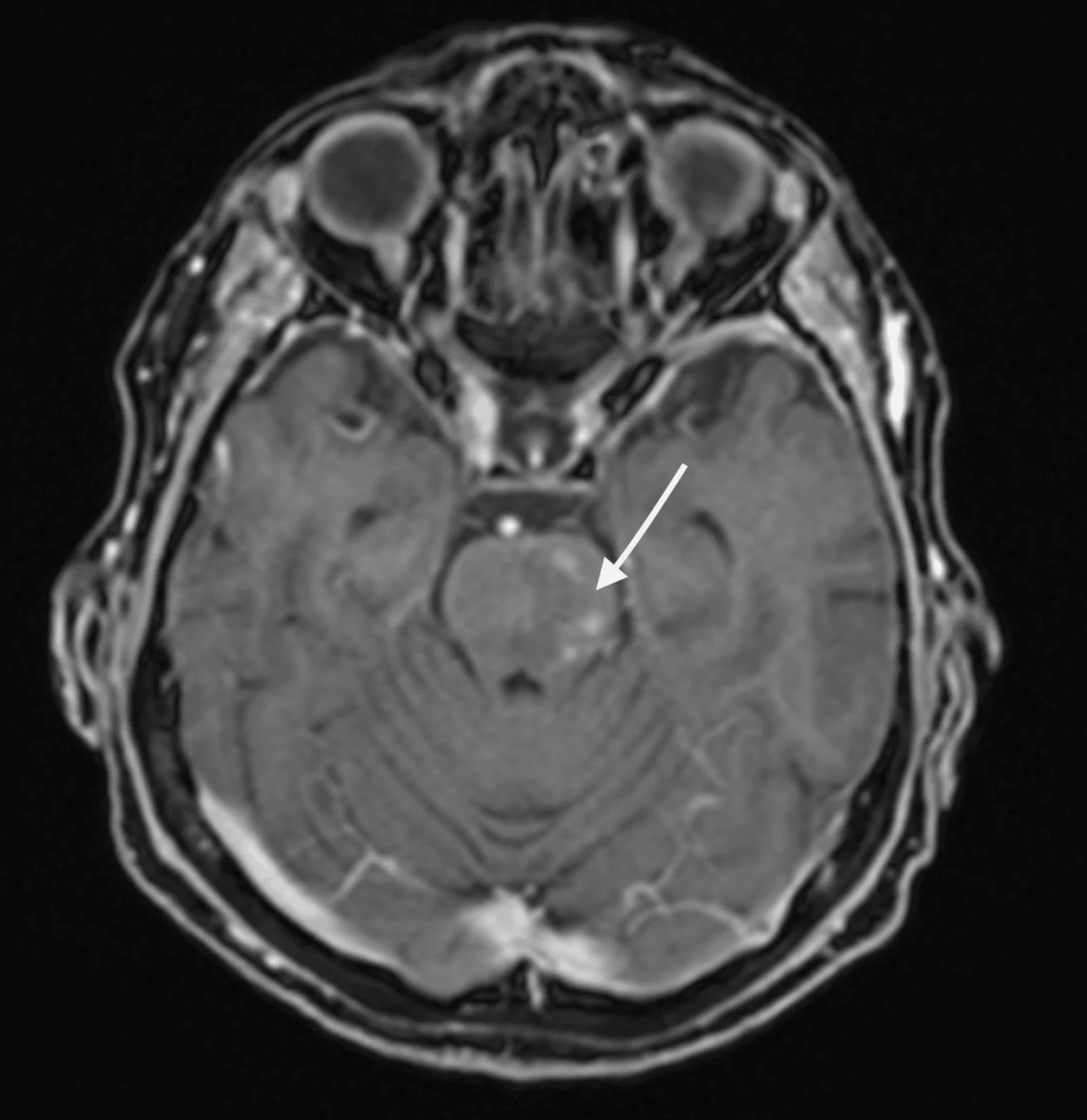
Axial T1-weighted post-contrast MRI demonstrating ill-defined patchy foci of enhancement within the left hemipons. The arrow indicates the patchy enhancing focus in the left dorsal pons.

**Figure 4:**
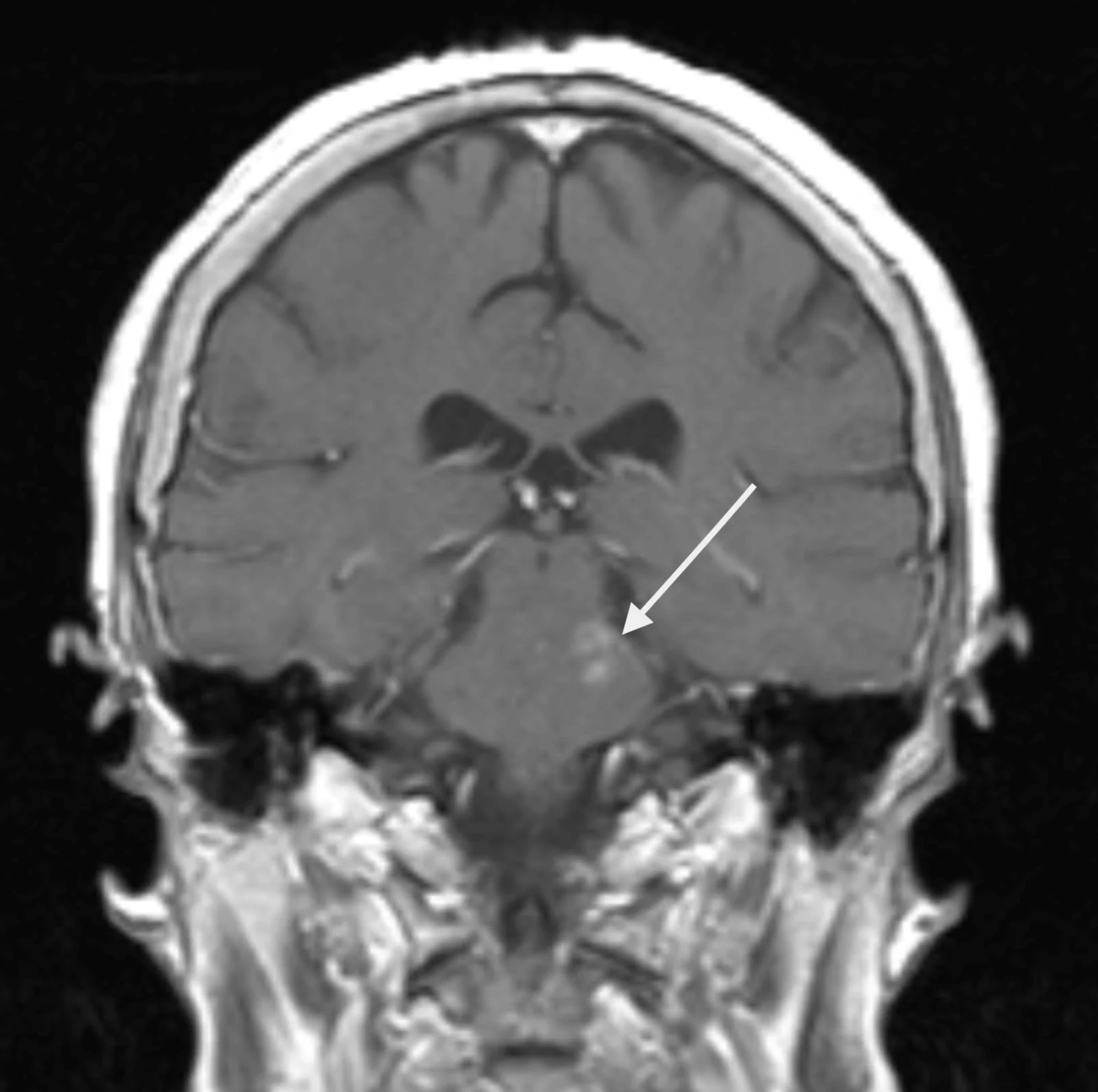
Coronal T1-weighted post-contrast MRI demonstrating corresponding patchy foci of contrast enhancement within the left hemipons. The arrow indicates the enhancing lesion in the left hemipons.

A lumbar puncture was performed to evaluate inflammatory, infectious, and neoplastic etiologies. Cerebrospinal fluid (CSF) analysis revealed 20 white blood cells/µL with lymphocytic predominance (77%), protein of 26.3 mg/dL, glucose of 52 mg/dL, and 1,389 red blood cells/µL, consistent with a traumatic tap. CSF cytology demonstrated no malignant cells, oligoclonal bands were absent, and intrathecal IgG synthesis was normal. A comprehensive CSF infectious polymerase chain reaction panel was negative.

Serologic evaluation for autoimmune demyelinating disease showed negative AQP4-IgG, while MOG-IgG was positive at a titer of 1:32 by live cell-based indirect immunofluorescence assay, supporting a diagnosis of MOGAD presenting as isolated brainstem encephalitis.

The patient received intravenous methylprednisolone 1 g daily for three consecutive days. Her diplopia improved substantially, although mild right-sided numbness persisted at discharge. Two weeks later, she experienced recurrent diplopia and was treated with oral prednisone 60 mg daily. A second recurrence occurred approximately one month after the initial presentation during steroid tapering, prompting initiation of mycophenolate mofetil as a steroid-sparing maintenance therapy.

At 2.5-month follow-up, repeat MRI demonstrated near-complete resolution of the pontine-midbrain T2-FLAIR lesion and complete disappearance of contrast enhancement (Figures 5-8), paralleling marked clinical recovery, and she remained free of further recurrences while tolerating mycophenolate mofetil without adverse effects.

**Figure 5:**
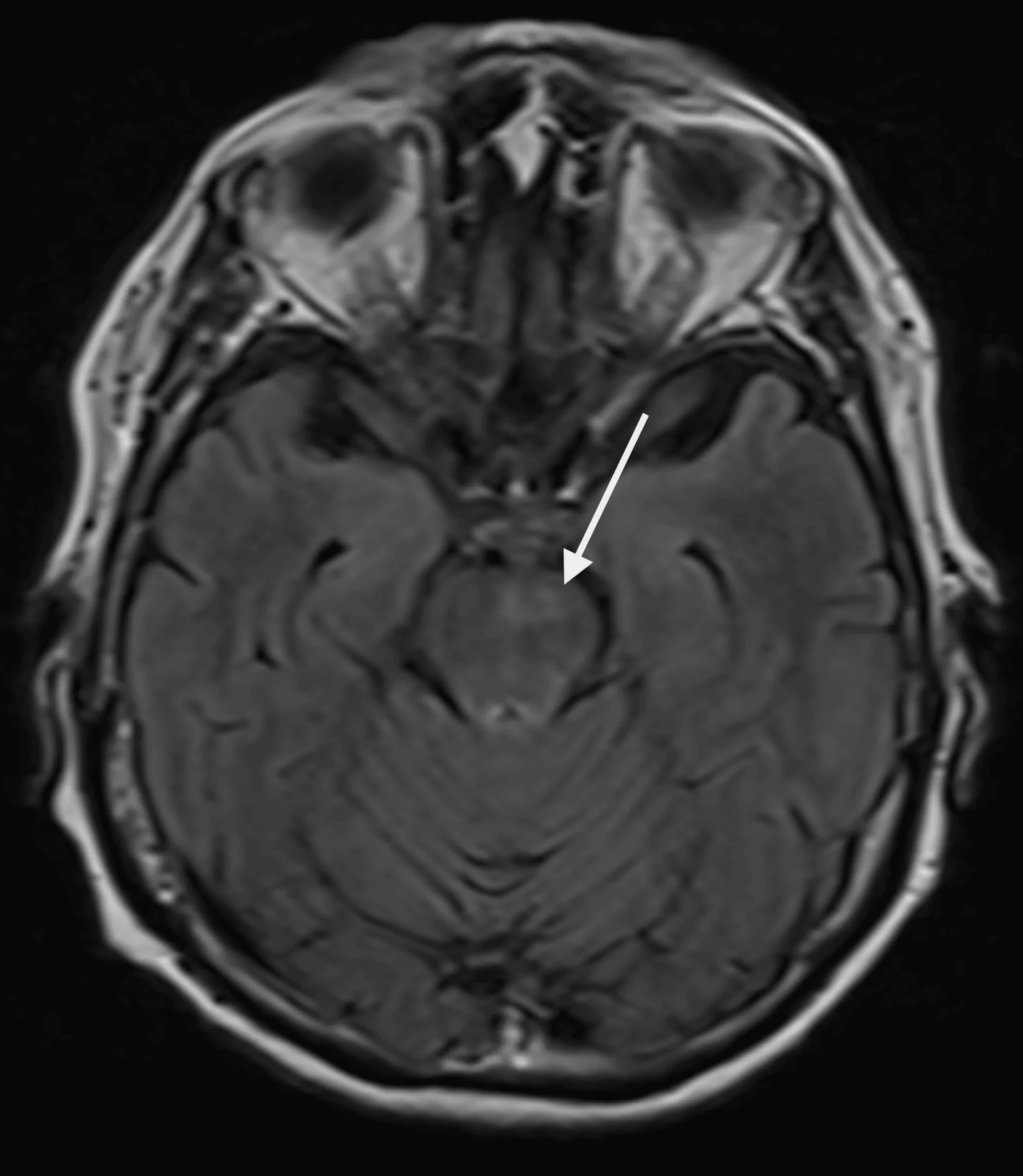
Follow-up axial T2-weighted fluid-attenuated inversion recovery (T2-FLAIR) MRI demonstrating near-complete resolution of the previously noted pontine hyperintensity. The arrow indicates the site of the previous left pontine lesion.

**Figure 6:**
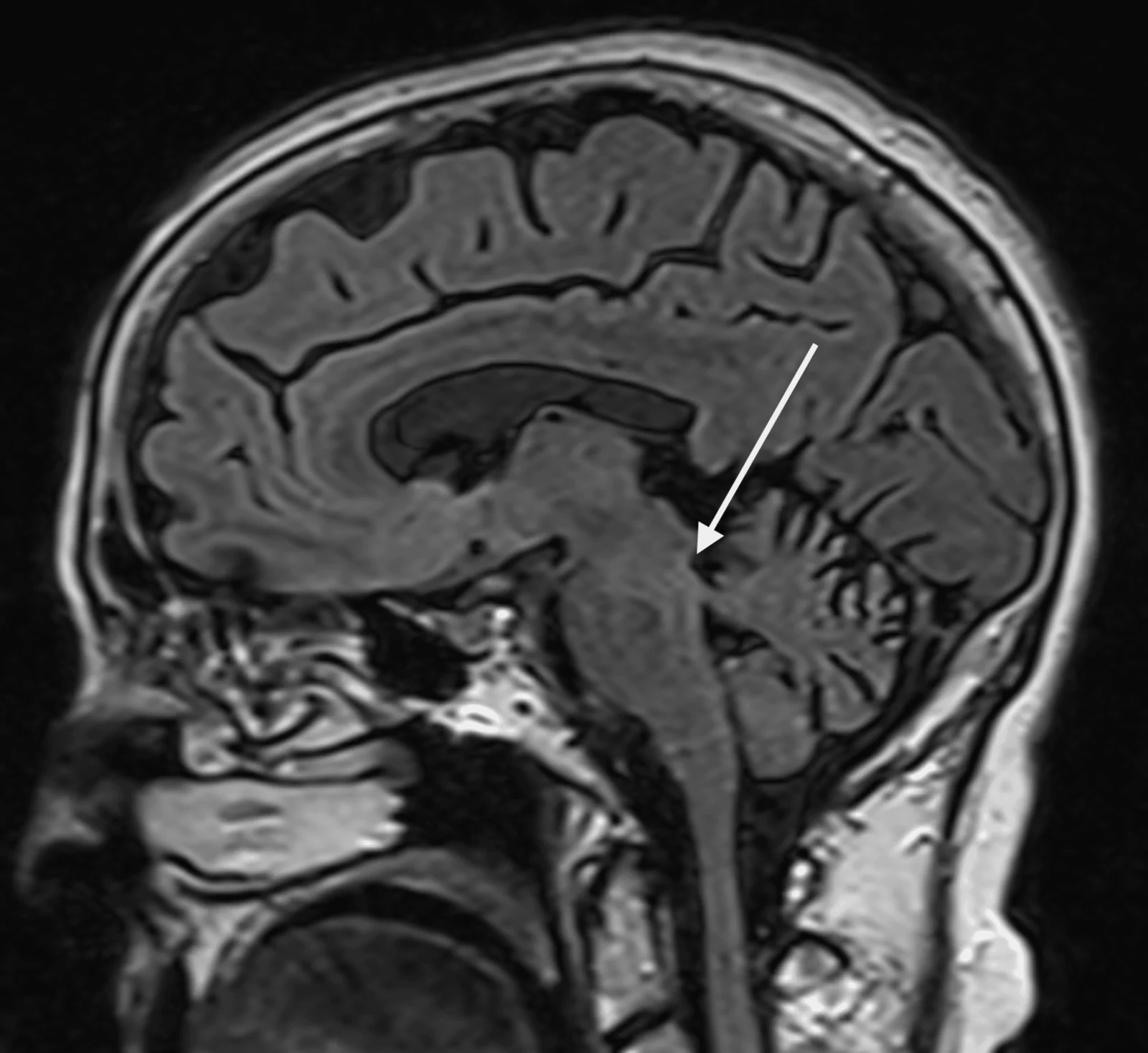
Follow-up sagittal T2-weighted fluid-attenuated inversion recovery (T2-FLAIR) MRI demonstrating near-complete resolution of the previously identified upper pontine hyperintensity. The arrow indicates the site of the previous upper pontine lesion.

**Figure 7:**
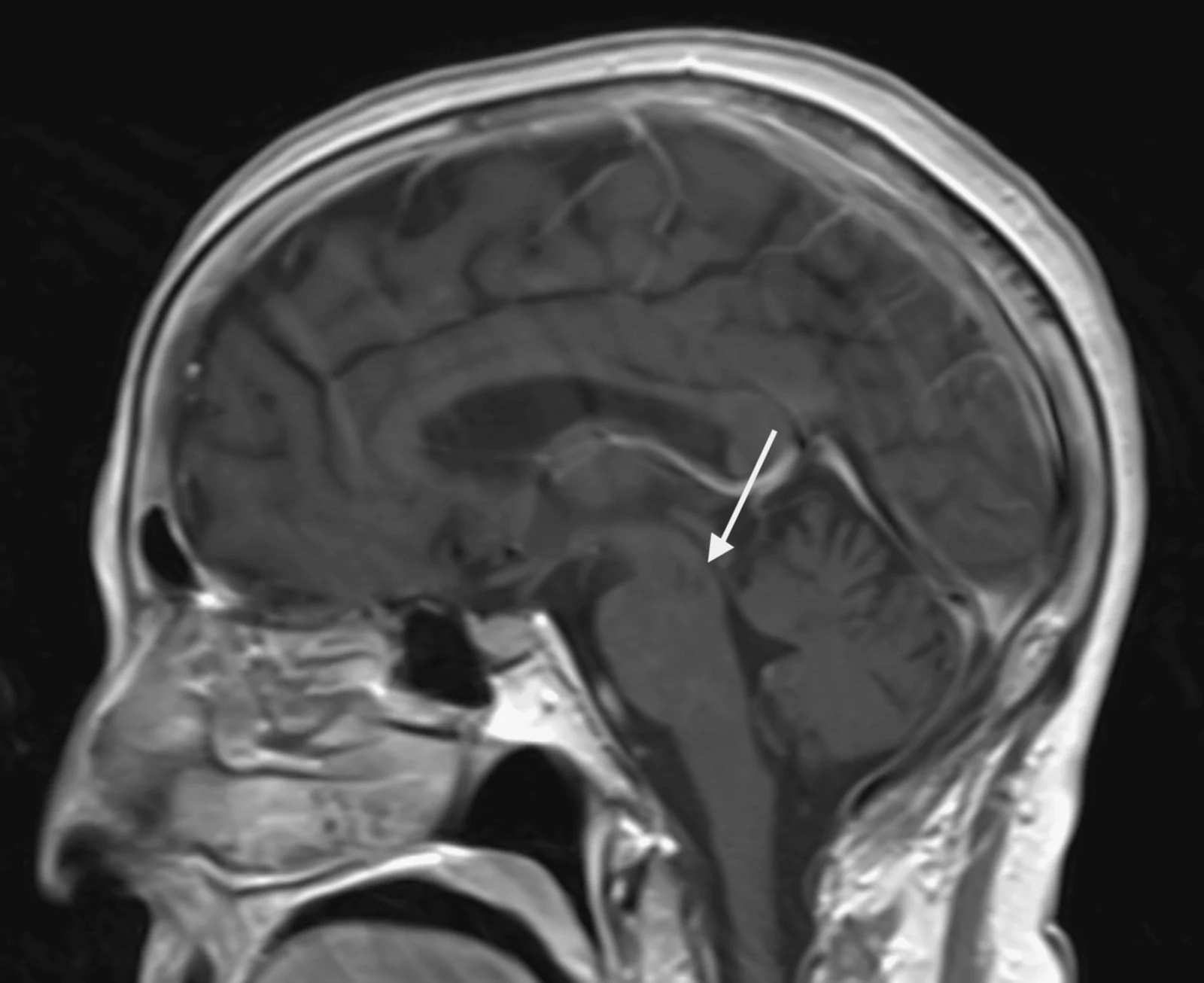
Follow-up sagittal T1-weighted post-contrast MRI demonstrating complete resolution of the previously observed abnormal pontine enhancement. The arrow indicates the site of the resolved upper pontine lesion.

**Figure 8:**
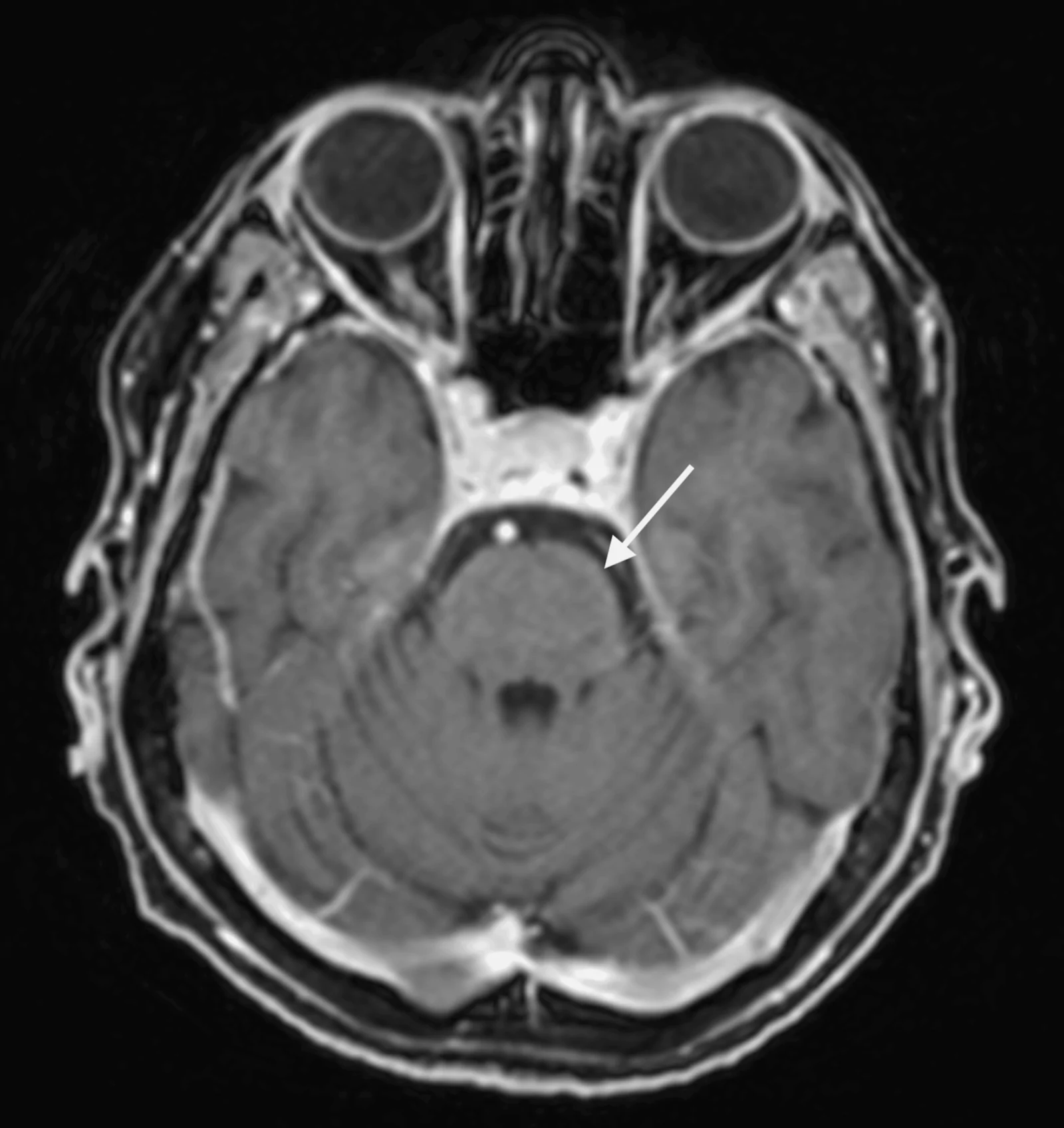
Follow-up axial T1-weighted post-contrast MRI demonstrating complete resolution of the previously observed abnormal pontine enhancement. The arrow indicates the site of the resolved left pontine lesion.

## Discussion

MOGAD is immunopathogenetically distinct from both MS and AQP4-IgG-positive NMOSD [15]. In the small number of biopsies reported, lesions have shown inflammatory demyelination resembling pattern II MS lesions, with perivascular and parenchymal T-cell infiltrates, macrophages containing myelin debris, complement deposition, and relative preservation of axons and astrocytes [1]. No biopsy was performed in our patient. This case is noteworthy for disease onset at 81 years, a brainstem-predominant lesion mimicking a neoplasm, and two early symptomatic recurrences after corticosteroid treatment that prompted maintenance immunosuppression.

MOGAD can occur across all age groups; in a UK cohort of 252 patients, onset ranged from 1 to 81 years [12], and clinical presentation and course vary with age at onset [22]. However, the clinical features of late-onset MOGAD remain less well characterized [22]. In one cohort, late-onset patients differed from younger patients in clinical presentation, MRI findings, and disease course, and were more likely to experience delayed diagnosis [22]. Our patient, who presented at 81 years of age, lies at the upper end of the age range reported in that UK cohort. Age-related clinical phenotypes and disease courses are summarized in Table 1.

**Table 1:** Age-related clinical presentations and disease course in myelin oligodendrocyte glycoprotein antibody-associated disease (MOGAD). Abbreviations: ADEM, acute disseminated encephalomyelitis; MOG, myelin oligodendrocyte glycoprotein; MOGAD, MOG antibody-associated disease; MS, multiple sclerosis. Source: Prepared by the authors based on published literature [10,20,22,23].

Age group	Clinical presentation	Disease course
Pediatric-onset	Acute disseminated encephalomyelitis (ADEM) is a prominent presentation among children with MOG antibodies [20].	Higher MOG-antibody titers were associated with recurrent non-MS episodes in children with acquired demyelinating syndromes [20].
Adult-onset	Optic neuritis or myelitis accounted for 90.9% of presenting syndromes; isolated brainstem or encephalopathy syndromes accounted for 6.6% [10].	The cumulative probability of a first relapse was 44.8% at 2 years and 61.8% at 5 years [10].
Late-onset (≥50 years)	Milder onset and more cerebral symptoms were reported compared with younger-onset patients in one cohort [22]. Brain involvement at onset was less frequent in late-onset than in younger-adult-onset patients in another cohort [23].	A monophasic course was more frequent in late-onset patients in both cohorts [22,23]. In the larger cohort, late onset was not significantly associated with time to first relapse but was associated with a higher risk of moderate disability at follow-up [23].

Brainstem involvement is less common in MOGAD than optic neuritis or myelitis, and isolated brainstem presentations are uncommon [10]. Pontine lesions, however, are among the recognized brain MRI findings in adults with MOGAD [10]. Brainstem lesions may mimic neoplastic or other inflammatory disorders, and MOGAD has been reported to mimic chronic lymphocytic inflammation with pontine perivascular enhancement responsive to steroids (CLIPPERS) [24-26]. CLIPPERS typically shows multiple small, punctate gadolinium-enhancing lesions involving the pons, midbrain, and cerebellum [26]. In contrast, the lesion in our patient was asymmetric and expansile, with patchy enhancement. Its near-complete resolution on T2-FLAIR imaging and complete resolution of enhancement following immunotherapy supported an inflammatory process. However, steroid responsiveness alone cannot distinguish MOGAD from CLIPPERS, and CLIPPERS-like presentations may also be associated with lymphoma [26]. Serum MOG-IgG testing should therefore be considered in patients with suspected CLIPPERS [26]. Recognizing these imaging mimics may help guide appropriate management and avoid unnecessary biopsy or oncological treatment [25,26].

The clinical course of MOGAD is variable and may be monophasic or relapsing [12]. Although MOGAD was initially considered predominantly monophasic, relapses occur in a substantial proportion of patients, with approximately 45-50% experiencing a relapse within two years in large cohorts [10,12]. Persistent MOG-IgG seropositivity has been associated with relapse, whereas patients who became seronegative remained relapse-free in one cohort [12]. Our patient experienced two early symptomatic recurrences, one two weeks after intravenous methylprednisolone and another during oral prednisone tapering. Recurrence during corticosteroid reduction or shortly after withdrawal has been described in MOGAD [11,16]. In one series of patients with relapsing MOGAD, 70% of episodes treated with oral prednisone were followed by relapse, particularly when the dose fell below 10 mg daily or within two months of treatment cessation [16]. Although the timing of our patient’s recurrences is consistent with this reported pattern, it does not establish whether they represented new relapses or recurrence of the initial attack. Mycophenolate mofetil was subsequently initiated, and the patient remained clinically stable at the 2.5-month follow-up. Immunosuppression extending beyond three months after the first attack has been associated with a lower relapse risk [12], and maintenance immunotherapy has been associated with reduced relapse rates in patients with relapsing disease [16]. However, the short follow-up in this case is insufficient to establish the long-term efficacy of mycophenolate.

In older adults, comorbidities may complicate the clinical picture, and diagnosis and treatment of MOGAD may be delayed [22]. Although onset in the ninth decade is rare, onset at 50 years or older is not uncommon [22,23], and MOGAD should be considered in older patients with atypical brainstem lesions, even when a neoplasm is suspected. Because steroid responsiveness does not exclude lymphoma in CLIPPERS-like presentations [26], diagnosis should rely on MOG-IgG testing using a cell-based assay with full-length human MOG, with confirmation in a second, methodologically different assay when results are positive in atypical presentations [15]. Under the 2023 International MOGAD Panel criteria, a low-positive titer may support the diagnosis in an AQP4-IgG-negative patient with a supporting clinical or MRI feature, such as an ill-defined pontine T2 lesion, as in our patient [27]. Maintenance immunosuppression may be considered for relapsing disease [14,16]. Although visual and motor outcomes are generally better in MOGAD than in AQP4-IgG-positive NMOSD [10], late-onset disease has been associated with greater disability at follow-up than disease with onset at 18-49 years [23], underscoring the importance of timely recognition and treatment.

This report has several limitations. The diagnosis rests on a low-positive MOG-IgG titer without histopathological confirmation, as no biopsy was performed. The neurological examination at presentation was incompletely documented, including gait assessment. Follow-up was limited to 2.5 months, which is insufficient to determine the long-term disease course or the effect of mycophenolate mofetil.

## Conclusions

MOGAD should be considered in the differential diagnosis of elderly patients presenting with atypical brainstem lesions, particularly when MRI features mimic neoplasm. Early recognition may help prevent unnecessary interventions, enable appropriate immunotherapy, and anticipate relapse risk.
